# Corneal endothelial and retinal microvascular changes in pseudoexfoliation syndrome: insights from specular microscopy and OCT angiography

**DOI:** 10.1186/s12886-025-04033-8

**Published:** 2025-04-11

**Authors:** Kursat Atalay, Ibrahim Kocak, Nihat Sayin, Busra Cirak

**Affiliations:** Kanuni Sultan Suleyman Training and Research Hospital, Eye Clinic, Istanbul, 34303 Atakent Mah. Turgut Ozal Blv. Kucukkcekmece Turkey

**Keywords:** Cornea endothelium, Specular microscope, OCTA, Optical coherence tomography, Pseudoexfoliation

## Abstract

**Purpose:**

To investigate the correlation between corneal specular microscopy (SM) and macular optical coherence tomography angiography (OCTA) data in patients with pseudoexfoliation syndrome (PXS).

**Methods:**

This was a cross-sectional, prospective study. Consecutive patients aged > 45 years with PXS or normal examination results were included. We examined cell density (CD), average cell area (AVG), coefficient of variation (CV), and hexagonality values from the SM. We collected 6 × 6 mm macular OCTA data with a quality score of 6/10 or higher.

**Results:**

Thirty-five eyes with PXS and 32 healthy eyes were evaluated. The CD, AVG, CV, and hexagonality for the PXS group were 2388.5 ± 368.8 cell/mm^2^, 435.0 ± 117.6 µm^2^, 28.9 ± 4.8%, and 67.6 ± 4.8%, respectively. The control group had the following values for CD, AVG, CV, and hexagonality: 2654.5 ± 269.8 cell/mm^2^, 380.4 ± 38.8 µm^2^, 29.8 ± 5.1%, and 67.1 ± 4.9%, respectively. No correlation was observed between OCTA and SM findings in the PXS group. We found a slight but significant link between CD and OCTA outer and total field estimations in the control group (*p* = 0.03 *r* = -0.25 and *p* = 0.02, *r* = 0.27, respectively). AVG and OCTA data in the control group indicated an association in the outer- and full-field analyses (*p* = 0.03 *r* = 0.25, *p* = 0.02, *r* = 0.27, respectively).

**Conclusion:**

Highlighting the observed lack of correlation between SM and macular OCTA findings in PXS patients in this study emphasizes the necessity of prioritizing early detection of anterior segment changes. The results indicate that immediate monitoring and intervention could reduce undesired consequences and maintain visual function in those affected.

## Introduction

Pseudoexfoliation syndrome (PXS) is characterized by the irregular deposition of pearl-like, granular, amyloid-like materials in many tissues and organs [1, 2]. It induces alterations in the physiological and anatomical features of the anterior segment of the eye, including the cornea, lens, zonules, trabecular meshwork, and iris [3–5]. PXS can induce alterations in both retinal vessels and systemic vascular structures [6–11]. In addition, similar cell pathways are shared during the embryonic development of the retinal vascular systems and corneal endothelium [12, 13].

The corneal endothelium is inspected using a specular light reflex with a slit lamp. More accurate measurements can be obtained with specular microscopes (SM), all of which are based on the technology introduced by Maurice [14]. The basic principle of a SM is to produce a slit of light that focuses on the corneal endothelium layer and, by viewing on a real-time monitor, handles the specularly (mirror-like) reflected light rays [15]. Endothelial cells are hexagonal in shape, arrested in the G1 phase of the cell cycle, and non-dividing in vivo under normal conditions [16, 17]. Though approximately 5000–6000 cell/mm^2^ cells are present at birth, the number gradually declines at a rate of 0.6%/year [18]. The accumulation of PXS and disruption of physiological activities can cause the corneal endothelium to decompensate, which can lead to keratopathy [19].

With the help of flowing blood, an optical coherence tomography angiography (OCTA) device can acquire images of ocular vessels by detecting motion contrast [20]. Although fluorescein angiography is still the best choice for retinal vascular disease evaluation, OCTA has the advantages of noninvasiveness and time efficiency, with the ability to yield three-dimensional retinal vasculature images [21]. The retinal vascular system is not immune to the pathophysiological effects of PXS, as demonstrated by numerous studies, most of which have dealt with OCTA [6, 7].

While previous studies have investigated SM and OCTA findings independently in PXS patients, the complex interplay between corneal endothelial changes and retinal vascular alterations remains largely unexplored. Understanding this relationship could provide valuable insights into the pathophysiology of PXS and guide the development of integrated diagnostic and management strategies.

## Methods

### General information and patient selection

This research was conducted at the Ophthalmology Department of the Kanuni Sultan Süleyman Training and Research Hospital between September 2023 and July 2024. The principles stated in the Declaration of Helsinki were obeyed for the conduct of this study, which was approved by the hospital ethics committee. The number of ethics committee permission is “KAEK/2023.12.203”. This was a hospital-based, randomized, non-interventional, prospective study. There is no clinical trial number that is applicable to this study. Informed consent was obtained from participants as part of the investigation protocol. The inclusion criteria covered patients over 45 years of age diagnosed with PXS or exhibiting normal examination results. The exclusion criteria included systemic disorders such as diabetes mellitus, cardiovascular problems, and previous ocular procedures or injuries that could distort the results.Patients who were non-smokers and had no previous eye injuries or surgeries were included in this study. Individuals with a history of optic neuritis and retinal illnesses were not included. The study included individuals whose spherical equivalent measurements fell within the range of + 3.00 D to -5.00 D. A comprehensive ophthalmological examination, which included a mydriatic retinal examination, was performed for each patient. For mydriatic assessment, a 0.1% tropicamide ophthalmic solution was used. One user (B.C.) performed the measurements using OCTA (Cirrius 5000 Carl Zeiss Meditec, Inc., Dublin, USA) and corneal SM (CEM-530 Nidek Co., Aichi, Japan) in compliance with the manufacturer’s instructions. Only OCTA measurements with a quality of 6/10 or higher were assessed.

### Diagnosis of pseudoexfoliation

Standardized diagnostic criteria established by Sternfeld and collegues was utilized to ensure the accurate identification of PXS [22]. Their manuscript outlines the diagnostic criteria for PXS. The patients with PXS were identified through the observation of characteristic pseudoexfoliation material on the anterior lens capsule and pupillary margin, lens blurring, and iris atrophy during a detailed slit-lamp examination.

### Instrumentation

#### Nidek CEM-530 specular microscope

Nidek CEM-530 (Nidek Co., Aichi, Japan) has the ability to take measures both manually and automatically; measurements taken in automatic mode were used in this investigation. Given a corneal radius of 7.8 mm, the device evaluates one point in the center, eight points at a distance of 1.3 mm from the center, and six points at a distance of 7.3 mm from the center. With an error margin of ± 10 μm, it is capable of measuring corneal thickness within a range of 300–1000 μm. This device analyzes the specular features of the endothelium, including average cell density (CD), average cell area (AVG), coefficient of variation (CV), and hexagonality (HEX).

#### Cirrus 5000 optical coherens tomography angiography

**Fig. 1:**
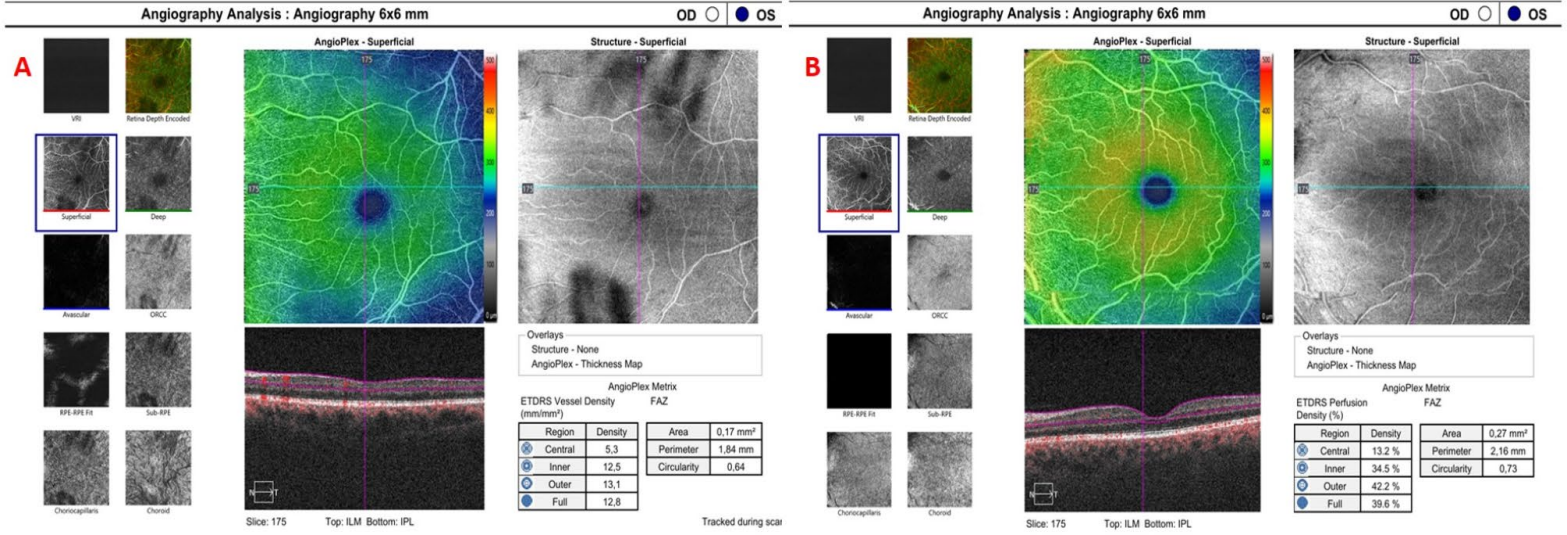
Optical coherence tomography angiography of one patient from the control group (**A**) and one from the pseudoexfoliation group (**B**).

The Cirrus 5000 device (Carl Zeiss Meditec, Inc., Dublin, USA), a spectral domain optical coherence tomography device, uses a superluminescent diode at 840 nm. The instrument gains 100,000 A-sections at depths from 2.0 to 2.9 mm within a second. Sections measuring 3 × 3, 6 × 6, 8 × 8, and 12 × 12 mm^2^ are available for OCTA assessment of the macula. This study relied on the OCTA data collected from 6 × 6 measurements. Figure 1 shows OCTA images of one control and one PXS patient. The AngioPlex Metrix measurements utilize OCTA criteria, such as the foveal avascular zone, perfusion density (ETDRS grid), and Vessel Density (ETDRS grid). This study investigated the superficial layer perfusion density of the macular region using information automatically generated by the OCT device. The values obtained in the central, inner, outer, and full areas of the test were evaluated.

### Statistics

The G-Power statistical software was used to conduct the power analysis for our study [23]. In the analysis, when the effect size was taken as 0.5, the α value was taken as 0.05 and the 1-β value was taken as 0.85, the number of samples to be included in the study was determined as at least 29. Statistical analyses were performed using SPSS for Windows (version 21.0, SPSS, Inc., Chicago, Illinois, USA). The Kolmogorov–Smirnov (K-S) method was employed to assess normal distribution, as it offers benefit in nonparametric data evaluation and serves as a versatile tool to test the goodness of fit for any continuous distribution. The chi-square test was used to compare categorical variables. The Student’s t-test was applied to analyze independent variables that are normally distributed. Kendall’s Tau-b was chosen for correlation analysis because some of the data, such as percentages, did not meet the assumptions of normality required for Pearson’s correlation. Mean ± Standard Deviation (mean ± SD) was used to show data with a normal distribution. Statistical significance was demonstrated by a p-value of less than 0.05.

## Results

Thirty-five eyes of 23 PXS patients were evaluated: 10 were male (43.5%) and 13 were female (56.5%). In the control group, 32 eyes of 20 patients were evaluated: 6 were male (30.0%) and 14 were female (70.0%). There was no statistical difference in the sex distribution between the two groups (*p* = 0.57). The PXS group had an average age of 66.5 ± 5.4 years. The control group had a mean age of 62.2 ± 8.3 years. There was no difference in the mean age between the groups (*p* = 0.059).

PXS glaucoma was present in 17 eyes of patients with PXS (48.6%), while only PXS findings were found in 18 eyes (51.4%). None of the patients in the control group was diagnosed with glaucoma. In the PXS group, the mean intraocular pressure was determined to be 18.03 ± 4.9 mmHg. The control group had an intraocular pressure (IOP) of 13.5 ± 2.1 mmHg on average. The mean IOP values of the PXS group were significantly higher than those of the control group (*p* < 0.00). The average measurements and comparison of the SM and OCTA results of the patients in the PXS group and the control group were examined and these results are shown in Table 1.

**Table 1:** The average measurements of the specular microscope and optical coherence tomography results for pseudoexfoliation syndrome and control group with their comparison

	PXS group (*n* = 35)				Control Group(*n* = 32)				
	Mean	SD	Minimum	Maximum	Mean	SD	Minimum	Maximum	P*
Cell Density (cells/mm^2^)	2388.5	368.8	956	3089	2654.5	269.8	2186	3167	*P* < 0.05
Average Cell Area (cells/mm^2^)	435.0	117.6	324	1046	380.4	38.8	316	457	*P* < 0.05
Cell size variation coefficient (%)	28.9	4.8	17	38	29.8	5.1	20	43	*P* = 0.48
Hexagonality (%)	67.6	4.8	56	77	67.1	4.9	56	75	*P* = 0.65
Corneal thickness (µm)	559.8	33.0	497	640	531.9	19.6	502	588	*P* < 0.05
Optic disk nerve fiber layer thickness (µm)	82.9	14.3	49	107	95.5	10.0	77	113	*P* < 0.05
Ganglion cell complex thickness (µm)	74.2	13.8	32	101	83.6	6.4	72	96	*P* < 0.05
OCTA^†^ Vessel Density Central (mm/mm^2^)	4.2	3.2	0.2	10.3	4.0	3.2	0.1	13.6	*P* = 0.8
OCTA^†^ Vessel Density Inner (mm/mm^2^)	11.1	5.6	1.8	19.2	10.8	4.6	1.7	18.1	*P* = 0.7
OCTA^†^ Vessel Density Outer (mm/mm^2^)	12.0	4.8	2.8	18.3	12.3	4.0	3.3	18.9	*P* = 0.7
OCTA^†^ Vessel Density Full (mm/mm^2^)	11.6	4.9	2.7	17.9	11.8	4.0	2.8	18.6	*P* = 0.8
OCTA^†^ Perfusion Central (%)	8.9	7.0	0.3	22.7	8.2	7.3	0.1	30.5	*P* = 0.59
OCTA^†^ Perfusion Inner(%)	25.5	13.3	3.8	45.7	24.0	11.8	3.4	43.8	*P* = 0.52
OCTA^†^ Perfusion Outer(%)	28.8	12.2	6.3	46.6	28.5	11.3	7.0	46.7	*P* = 0.85
OCTA^†^ Perfusion Full(%)	27.5	12.1	6.1	45.2	26.9	11.0	6.0	45.6	*P* = 0.76

^†^ Optic Coherence Tomography Angiography is abbreviated as OCTA

*P denotes independent sample T-test

The PXS group had an average central macular thickness (CMT) of 256.3 ± 28.00 μm, while the control group had an average of 261.4 ± 24.03 μm. There was no statistically significant difference between the CMT averages of the two groups (*p* = 0.37).

The relationship between corneal endothelial readings obtained by SM and macular perfusion values of PXS cases is shown in Table 2. It is evident from this table that there is no statistically significant correlation between the data collected from the patients.

**Table 2:** Correlation analysis of pseudoexfoliation syndrome group for specular microscopy and optical coherence tomography angiography

		OCTA^†^ Perfusion Central (%)	OCTA^†^ Perfusion Inner (%)	OCTA^†^ Perfusion Outer (%)	OCTA^†^ Perfusion Full (%)
Cell Density (cells/mm^2^)	r*	0.11	0.09	0.14	0.14
	P*	0.31	0.40	0.21	0.22
Average Cell Area (cells/mm^2^)	r*	-0.12	-0.10	-0.14	-0.14
	P*	0.30	0.39	0.21	0.22
Cell size variation coefficient (%)	r*	0.05	0.09	0.05	0.06
	P*	0.64	0.44	0.64	0.59
Hexagonality (%)	r*	0.04	0.05	0.09	0.09
	P*	0.69	0.62	0.41	0.44

*P denotes Kendall Tau b statistical significance level, r denotes correlation coefficient

^†^ Optic Coherence Tomography Angiography is abbreviated as OCTA

Table 3 demonstrates the association between the macular perfusion values evaluated by OCTA and the SM findings from the control group. This table demonstrates a significant correlation between cell density, AVG, and superficial macular perfusion density in the outer and full layers.

**Table 3:** Correlation analysis of control group for specular microscopy and optical coherence tomography angiography

		OCTA^†^ Perfusion Central (%)	OCTA^†^ Perfusion Inner (%)	OCTA^†^ Perfusion Outer (%)	OCTA^†^ Perfusion Full (%)
Cell Density (cells/mm^2^)	r*	-0.08	-0.19	-0.25	0.27
	P*	0.51	0.11	0.03	0.02
Average Cell Area (cells/mm^2^)	r*	0.08	0.19	0.25	0.27
	P*	0.50	0.11	0.03	0.02
Cell size variation coefficient (%)	r*	-0.10	-0.08	-0.07	-0.07
	P*	0.40	0.51	0.55	0.54
Hexagonality (%)	r*	0.11	0.06	0.14	0.11
	P*	0.38	0.60	0.26	0.35

*P denotes Kendall Tau b statistical significance level, r denotes correlation coefficient

^†^ Optic Coherence Tomography Angiography is abbreviated as OCTA

## Discussion

The present study demonstrated that patients with PXS showed substantial reductions in corneal endothelial cell density, average cell area, and an increment in central corneal thickness when compared to the control group. Notably, no correlation was observed between SM and macular OCTA findings in the PXS group, whereas a weak but statistically significant correlation was present in the control group. These results suggest that anterior segment changes occur earlier than retinal vascular alterations in PXS, highlighting the importance of early detection and monitoring to prevent disease progression. The anterior portion of the eye, including the cornea, lens, zonular fibers, trabecular meshwork, and iris, undergoes changes in anatomy and physiology due to PXS [3–5]. Evidence suggests that PXS can affect the retina and circulatory system as a whole [6–11, 24]. Despite studies examining the corneal endothelial layer of PXS patients and focusing on the deterioration of SM or changes in the retinal vascular system, there is a scarcity of studies examining the relationship between SM and macular OCTA changes in the current literature.

A single layer of in vivo non-dividing, hexagonal cells compose the corneal endothelium, which decreases in number at approximately 0.6%/year. This results in a decreased number of cells and compensatory morphological alterations. In contrast to controls, PXS patients showed considerable decreases in corneal endothelial cell density, AVG, and increase in CCT, according to this study. Multiple factors, including the direct accumulation of pseudoexfoliative material, oxidative stress, impaired aqueous humor dynamics, chronic inflammation, and genetic predispositions, may contribute to these results [3, 25]. Tomaszewski et al. reported that PXS patients exhibited significantly reduced corneal endothelial cell density (2297 ± 359 cell/mm^2^) compared to normal patients (2503 ± 262 cell/mm^2^) [3]. Bozkurt and colleagues used confocal microscopy to analyze corneal endothelial cell density data from individuals with PXS, pseudoexfoliation glaucoma, and senile cataracts. They found no significant difference in endothelial cell density among the three groups. Nevertheless, there were no significant statistical disparities in relation to polymegathism and pleomorphism [4]. De Juan-Marcos et al. examined patients with PXS, pseudoexfoliation glaucoma, and POAG, and normal patients [5]. They measured significantly lower values in terms of endothelial cell density in the PXS (2346 ± 256.88 cell/mm^2^) and POAG groups (2294 ± 235.82 cell/mm^2^) than in normal patients (2565 ± 270.17 cell/mm^2^). In the same study, they found that the percentage of hexagonality was lower in the PXS and POAG groups. In line with prior investigations, the analysis of this study demonstrated that PEX patients had a significantly lower endothelial cell density than the control group. However, the hexagonality of the endothelial cells was not significantly different between the PXS and control groups. Wang et al. found a decrease in corneal endothelial cell density, but no significant change in hexagonality and coefficient of variation in Chinese patients [26]. Quiroga et al. evaluated patients in Paraguay who had PXS cataracts [27]. They discovered that endothelial cell density decreased, but there was no significant change in hexagonality [27]. Naumann et al. demonstrated that PXS causes severe damage to the corneal endothelium and defined these changes as non-Fuchs endotheliopathy using histopathologic methods [19]. Similarly, Zheng et al. suggested that the difference in severity between the eyes with PXS findings and the other eye might be due to the unequal staging of unilateral disease in the Japanese population [28]. These results suggest that the decrease in CD in PXS begins much earlier than the changes in cell morphology, and the compensatory effects developing at the intracellular level may have occurred earlier than morphological compensation. Current literature is not conclusive, as some studies show a decrement but others an increment about the CCT changes in PXS patients [29]. Yuksel and colleagues find an increased CCT level with confocal microscopy in pseudoexfoliation glaucoma patients [30]. They have speculated this finding with progressive pathologic alterations in the corneal endothelium can affect stromal hydration, which in turn increases CCT [30]. The present study included patients with pseudoexfoliation glaucoma, which may have led us to obtain a result similar to the findings of Yüksel et al. Initial findings of this study suggest focusing anterior segment evaluations in PXS patients could enhance early disease management. Due to the substantial changes noted in corneal endothelial measures, ophthalmologists might consider earlier and more regular assessments of anterior segment abnormalities in PXS patients.

Çınar et al. conducted a study using the Optovue AngioVue (Optovue^®^ 2800 Bayview Dr., Fremont, CA 94538) to assess 38 eyes of PXS patients and 35 eyes of normal patients [6]. In their study that compared to healthy control eyes, PXS eyes showed decreased total, parafoveal, and foveal vascular densities in the superficial capillary plexus of the retina. In the study by Çınar et al., the choroidal thickness was much thinner in people with PXS [6]. Köse et al. examined the changes in the peripapillary and macular regions of normal, POAG, PXS, and OHT patients with OCTA and found a statistically significant difference between all groups in the superficial plexus [7]. They found a significant difference in superficial plexus whole image vessel density (wiVD) measurements between the POAG and pseudoexfoliation glaucoma groups. The devices used to obtain OCTA measurements in the previous studies differed. This may account for the discrepancies across this study’s observations and the existing literature. In a study conducted by Cornelius et al., the perifoveal perfusion densities of patients with pseudoexfoliation glaucoma and POAG were examined using the Cirrus OCT-5000 device. The superior and average perfusion densities in their study were lower in pseudoexfoliation glaucoma patients [8]. When we compared the perfusion densities of the OCTA examination between the PXS and control patients, no significant difference was observed between the two groups. This finding is different from the results of Cornelius et al. In this study, 16 (32.7%) patients with PXS did not have glaucoma. Aditionaly, Cornelius et al. included patients with prior cataract or glaucoma surgery and diabetes in their analysis. Thus, patient characteristics may have an impact on differences found between the present study and Cornelius and colleagues’ study.

When concomitant factors such as open-angle glaucoma, age, sex, smoking, diabetes, steroid use, myopia, and socioeconomic status are controlled, the Blue Mountains Eye Study showed that PXS patients develop cataracts, especially of the nuclear type, and undergo cataract surgery earlier than normal individuals [31]. This is an important finding because patients with PXS may show earlier functional and structural deterioration at the anterior segment. There is a scarcity in the current literature examining longitudinal retinal vascular changes in patients with PXS, but Lee et al. examined the thinning of the ganglion cell inner plexiform layer in normal, open-angle glaucoma, and PXS patients in their trend-based study [32]. They showed that the ganglion cell layer decreased significantly in all groups, and most rapidly in patients with PXS. The current study also showed a significantly decreased number of ganglion cell complexes in patients with PXS. In the present study, a statistically significant correlation was seen between the data on corneal endothelial cells and the perfusion data of the macula in normal individuals; however, this relationship was not identified in patients with PXS. Proper perfusion of the macula allows the ganglion cell complex to maintain its function in a healthy manner. The findings highlight a significant gap in understanding the temporal dynamics between changes in the anterior and posterior segments in PXS. Future longitudinal works may aim to clarify the sequence of pathological changes to assess whether interventions addressing early anterior segment abnormalities can delay or halt subsequent retinal involvement. In this study, although there was no statistical difference in perfusion between the PXS patients and the control group, the deterioration of SM findings and the deterioration of the correlation with OCTA findings suggest that pathologies in the anterior segment occur much earlier than in the retinal vascular region.

Although the present study offers significant insights, numerous limitations must be considered in subsequent research. The cross-sectional design restricts the capacity of this research to deduce causality or temporal links between alterations in the anterior and posterior segments. Moreover, the possible impact of confounding variables, including glaucoma drug usage and systemic vascular health, necessitates additional examination. Addressing these limitations through meticulously designed longitudinal investigations would be crucial to thoroughly clarify the clinical and neurobiological implications of these findings.

## Conclusion

In this study, no correlation was observed between SM and superficial retinal OCTA data in patients with PXS. Changes in corneal findings have been observed in patients undergoing PXS. Long-term randomized studies are needed to examine the timing of the development of PXS-related changes and the interaction between different eye tissues.

## Data Availability

The data that support the findings of this study are not openly available due to reasons of sensitivity and are available from the corresponding author upon reasonable request.
